# A Rare Case of Cephalosporin Anaphylaxis-Induced Reverse Takotsubo

**DOI:** 10.7759/cureus.63987

**Published:** 2024-07-06

**Authors:** Omer Farooq, Roger Lin, Benjamin Easow, Paarmit Chhabra, Paramjit Kaur

**Affiliations:** 1 Internal Medicine, Southeast Health Medical Center, Dothan, USA

**Keywords:** takotsubo cardiomyopathy, cephalosporin anaphylaxis, heart failure with reduced ejection fraction, allergy and anaphylaxis, reverse takotsubo

## Abstract

This case report details an unusual occurrence of reverse takotsubo induced by cefazolin anaphylaxis. While anaphylactic reactions typically manifest with hypotension and bronchospasm, the development of takotsubo is a rare outcome. The patient experienced an episode of cefazolin-induced anaphylaxis during elective shoulder surgery, subsequently developing reverse takotsubo cardiomyopathy (rTTC) during her hospitalization. Initial testing showed a reduced heart function, with an ejection fraction (EF) dropping to 32% from a previously normal EF exceeding 50%. However, a follow-up heart catheterization three weeks later revealed a return to normal heart function. The patient received appropriate management for heart failure. By emphasizing the nuanced features and symptoms, we aim to enhance the recognition and management of this condition. Sharing such cases contributes to the medical community's knowledge and facilitates the advancement of strategies for diagnosing and managing anaphylaxis-induced reverse takotsubo.

## Introduction

Sato and Dote in 1990 and 1991 introduced the term takotsubo (tako = octopus, tsubo = a pot) to describe the left ventricular silhouette during systole in five patients presenting with clinical features of myocardial infarction but without obstructive coronary artery disease [1]. Takotsubo syndrome was first diagnosed in Hiroshima City Hospital Japan in 1983 [2]. The prevalence of estimated incidence is 2% among all troponin-positive patients with suspected ACS and up to 10% if only women are considered. Also known as takotsubo syndrome, it entails acute reversible left ventricular dysfunction typified by apical ballooning. More than 85% of the patients with TTC are said to be postmenopausal women (aged 65-70 years), therefore suggesting a possible hormonal response. In Western countries, there is a female-to-male ratio of 9:1 [3]. This non-ischemic cardiomyopathy is characterized by transient left ventricular systolic dysfunction and electrocardiogram alterations in the absence of coronary artery disease obstruction. Typically, it is a reversible condition, with patients generally experiencing full recovery.

Among various variants of takotsubo syndrome, one form is reverse takotsubo, where basal hypokinesis replaces the typical apical hypokinesis. Symptoms of reverse takotsubo differ from those of typical apical takotsubo, with patients exhibiting less pulmonary edema, dyspnea, and cardiogenic shock. This variance in clinical presentation suggests that differences in symptoms coincide with potential hemodynamic alterations caused by regional wall motion abnormality locations. The occurrence of inverted takotsubo cardiomyopathy (TTC) in younger patients may be attributed to a higher abundance of adrenoreceptors at the heart's base compared to the apex in older patients [4]. Common factors contributing to takotsubo syndrome include excess catecholamines, microvascular dysfunction, coronary artery spasms, and left ventricular outflow tract obstruction.

## Case presentation

A 66-year-old female, with a complex medical history encompassing coronary artery disease (CAD), atrial fibrillation with rapid ventricular response (AFib with RVR), pulmonary hypertension, sleep apnea, bipolar disorder, dyslipidemia, and hypertension, presented as a transfer patient due to suspected anaphylactic shock. Despite a history of multiple cardiac stents, the recent heart catheterization in 2021 revealed a patent left anterior descending artery (LAD) and an ejection fraction (EF) greater than 50%, but chronic apical hypokinesis secondary to prior myocardial ischemia.

Scheduled for elective reverse total shoulder arthroplasty, the patient, known for an anaphylactic reaction to penicillin, received cardiac clearance. However, following preoperative intravenous cefazolin administration, she experienced immediate respiratory distress. Her vitals were as follows: respiratory rate of 34, SPO_2_ of 82%, heart rate of 130, and blood pressure of 80/40. Despite interventions, including epinephrine, steroids, and Benadryl, her symptoms persisted, leading to emergent intubation due to hypoxic respiratory failure. With hypotension and tachycardia, an epinephrine infusion was initiated. Initial troponin I levels were elevated at 2,167, trending down subsequently. Diagnosis revealed non-ST elevation myocardial infarction (NSTEMI) type 2 from myocardial stunning due to anaphylactic shock.

**Figure 1:**
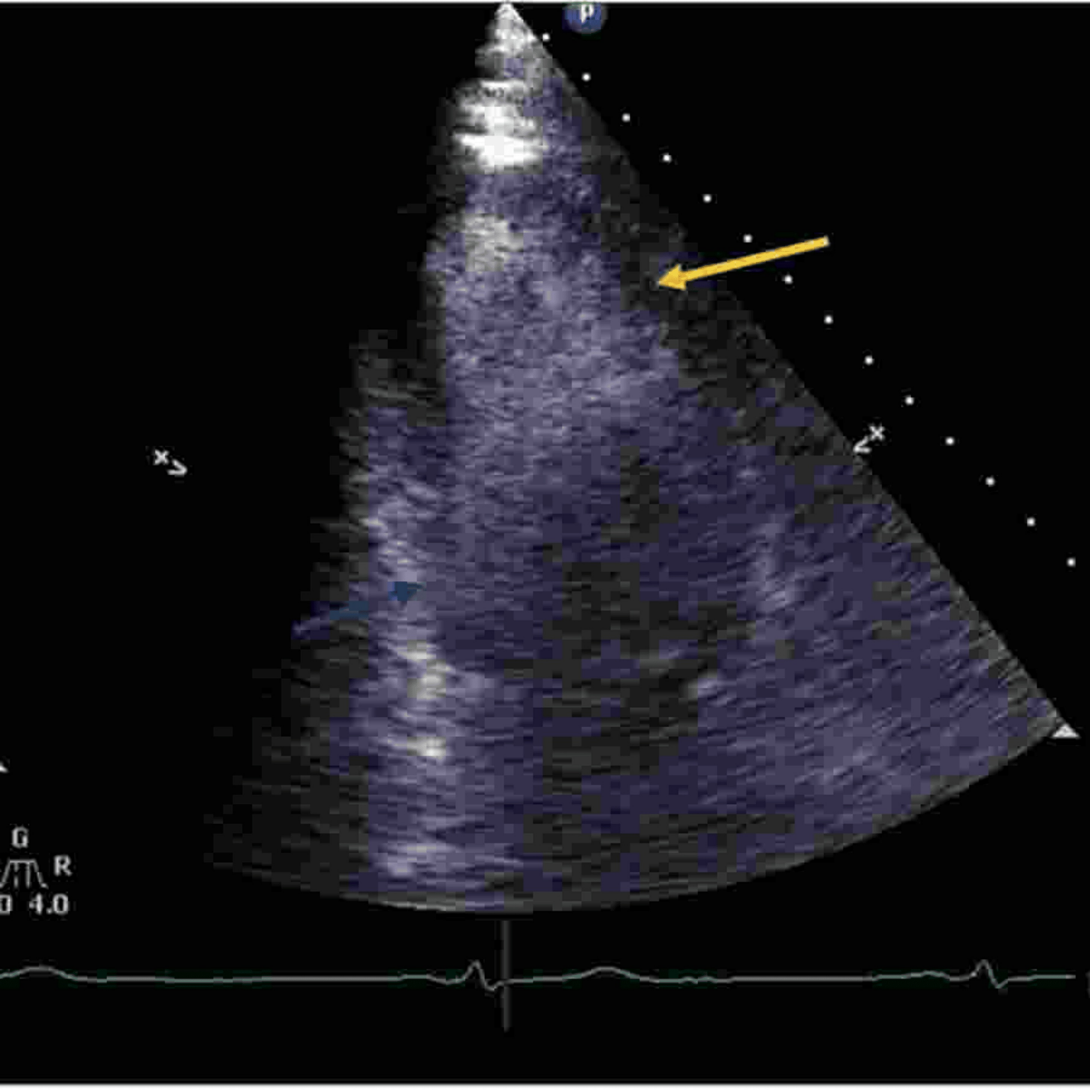
In this contrast TTE apical view of the left ventricle during systole, the yellow arrow indicates apical contraction of LV during systole, but at the same time blue arrow shows hypokinesis of the base of LV. There is some baseline chronic apical hypokinesis secondary to previous myocardial ischemia, but hypokinesis of the base of LV is prominent. LV: Left Ventricle, TTE: Transthoracic Echocardiogram

**Figure 2:**
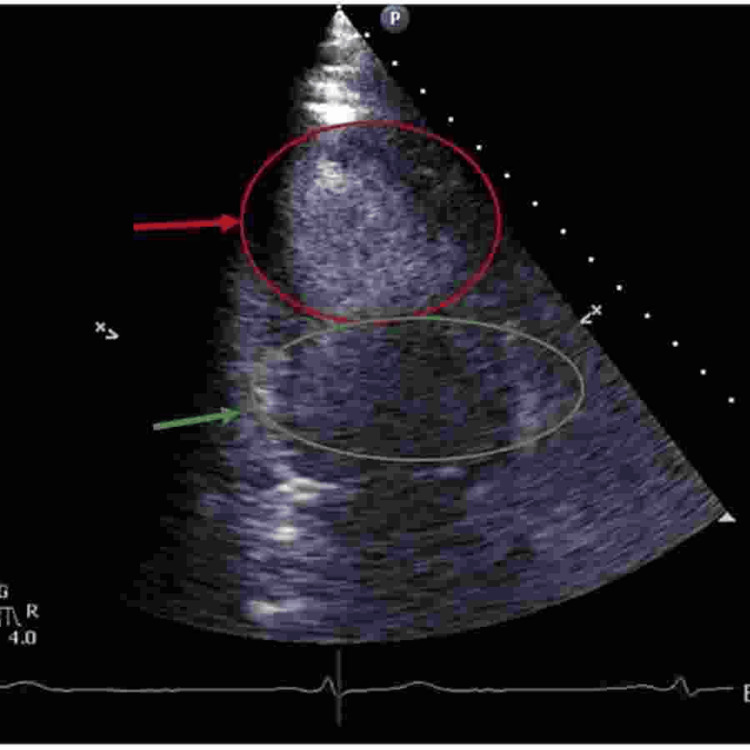
This is the same contrast TTE apical view of the left ventricle during systole; the red arrow indicates apical contraction of LV during systole, and the green arrow shows hypokinesis of the base of LV. LV: Left Ventricle, TTE: Transthoracic Echocardiogram

Transthoracic echocardiogram (TTE) demonstrated reverse takotsubo with an EF of 32% and grade 2 diastolic dysfunction. Figures 1-2 show the reverse takotsubo in the systolic phase. Despite a suspected pulmonary embolism (PE), ruled out by contrast CT chest, the patient experienced metabolic acidosis, later resolved with IV bicarbonate. Hospitalization complications included AKI secondary to hypotension, resolving with continuous IV fluids. After a rapid improvement, the patient was extubated within a day and discharged home after a four-day hospital stay.

Upon discharge, she was prescribed medications, including Lopressor 25 mg daily, Eliquis 5 mg twice daily, Aldactone 12.5 mg daily, aspirin 81 mg, EPI pen, and Metformin 500 mg daily. A cardiology follow-up three weeks later, including a repeat heart catheterization, revealed patent coronary arteries and a baseline EF exceeding 50%, indicating significant recovery from the acute episode.

## Discussion

Various presentations of left ventricular (LV) dysfunction in takotsubo cardiomyopathy (TTC) have been documented, encompassing the well-known apical form, as well as midventricular, basal (or inverted), and localized variations. In this case, we propose that several characteristics contribute to the manifestation of the basal variation of TTC, known as reverse TTC (rTTC). Emerging evidence in the context of TTC underscores the significant role of microvascular dysfunction [4]. Differences in susceptibility to microvascular spasms between TTC and rTTC patients, as seen in this case involving underlying coronary artery disease and multiple stent placements, may explain the unique basal hyperkinesis and apical sparing in rTTC, deviating from the typical apical ballooning seen in TTC.

Moreover, this case involves preexisting anterior apical hypokinesis attributed to a prior myocardial infarction. This condition may predispose the patient to rTTC, diverging from the classical form because of the inherent myocardial vulnerability and altering stress response patterns. The concept of regional myocardial dysfunction influencing atypical presentations of TTC is proposed, emphasizing the need for further research to elucidate underlying mechanisms and clinical significance.

Recent literature on rTTC triggered by anaphylaxis suggests a connection to exogenous epinephrine administration [5-6]. Elevated epinephrine levels in patients with rTTC, alongside the differing neurochemical profiles between TTC and rTTC patients, support its etiological role [7]. In this case, the patient received an epinephrine drip in the ICU, but the intricate interaction of various cytokines in anaphylaxis pathophysiology hints at potential mechanisms influencing rTTC. This nuanced understanding prompts further research into the cytokine-mediated dynamics of rTTC in anaphylactic contexts.

This case report expands the spectrum of rTTC triggers to include anaphylaxis, emphasizing the unpredictable nature of takotsubo syndrome and its variants. It underscores the importance of considering rTTC in patients with acute cardiac symptoms under significant stressors, including anaphylaxis, even without the typical ACS risk factors. Additionally, it highlights the necessity for awareness of drug allergies and the potential for severe reactions, including impacts on cardiac function. Patients known to have allergies to medications should be provided with a medical card upon discharge to help minimize the risk of anaphylactic reactions.

## Conclusions

In conclusion, this case report sheds light on the rare occurrence of reverse takotsubo syndrome triggered by cefazolin anaphylaxis, presenting a unique manifestation within the spectrum of anaphylactic reactions. It underscores the unpredictability of takotsubo syndrome and its variants, particularly in the context of unusual triggers such as anaphylaxis. Moreover, the case emphasizes the importance of considering rTTC in patients with acute cardiac symptoms, even without the typical risk factors for acute coronary syndrome. Additionally, it highlights the critical need for healthcare providers to be vigilant about drug allergies and their potentially severe impact on cardiac function. This case contributes to the growing body of knowledge about reverse takotsubo syndrome and underscores the importance of continued research and awareness in diagnosing and managing this condition effectively.
